# Low Molecular Weight and High Deacetylation Degree Chitosan Batch Alleviates Pathogenesis, Toxin Accumulation, and *Fusarium* Gene Regulation in Barley Leaf Pathosystem

**DOI:** 10.3390/ijms241612894

**Published:** 2023-08-17

**Authors:** Pawel Poznanski, Amir Hameed, Marta Dmochowska-Boguta, Marcin Bryla, Waclaw Orczyk

**Affiliations:** 1 Plant Breeding and Acclimatization Institute—National Research Institute, Radzikow, 05-870 Blonie, Poland; p.poznanski@ihar.edu.pl (P.P.); a.hameed@ihar.edu.pl (A.H.);; 2 Professor Waclaw Dabrowski Institute of Agricultural and Food Biotechnology—State Research Institute, Rakowiecka 36, 02-532 Warsaw, Poland; marcin.bryla@ibprs.pl

**Keywords:** antifungal activity, chitosan, disease control, fungal, *Fusarium* transcriptomic, molecular targets for *Fusarium* control, *Fusarium* mycotoxins, plant–pathogen interaction, plant protection strategies

## Abstract

*Fusarium graminearum* is a cosmopolitan fungal pathogen that destroys cereal production, in terms of loss of yield and grain contamination with mycotoxins, worldwide. Chitosan is a natural biopolymer abundant in the environment with proven antifungal properties that also acts as a plant immunity elicitor. Despite a number of articles, there is a lack of systematic comparison of antifungal activity of diverse batches of chitosan. The current study aimed to test the inhibitory effects of a collection of diverse chitosan samples on the growth and production of *F. graminearum* toxins, validated by changes in the *Fusarium* transcriptome. Experiments included testing antifungal activity of different chitosan samples, the application of the best performing one in vitro to investigate the impact on *F. graminearum* growth, followed by analyzing its effect on *Fusarium* toxins accumulation, and *Fusarium* transcriptomics in the barley leaf pathosystem. Confirmatory antifungal assays revealed that CS_10, a specific batch of chitosan, retarded *Fusarium* growth with an application concentration of 200 ppm, significantly reducing toxin synthesis and disease symptoms in *Fusarium*-inoculated barley leaves. RNA-Seq analysis of *F. graminearum* in barley leaf pathosystem exposed to CS_10 showed a list of differentially expressed genes involved in redox balance, cell respiration, nutrient transport, cell wall degradation enzymes, ergosterol biosynthesis, and trichothecenes production. The genes functioning in these essential pathways are discussed and assigned as critical checkpoints to control *Fusarium* infections. The results suggest some important molecular targets in *F. graminearum* that may be suitable in gene-specific targeting or transgene-free methods, such as spray-induced gene silencing during host-pathogen interactions.

## 1. Introduction

*Fusarium graminearum* (*Fg*) is a fungal pathogen that infects cereals, such as wheat, barley, maize, and other food crops around the world. It causes a disease called Fusarium head blight (FHB), also known as scab, which leads to significant yield losses and reduces grain quality [1]. FHB occurs when fungal spores are dispersed by wind or rain and land on cereal heads. The fungus then grows on the host and symptoms include bleached or discolored heads, lightweight and shrunken grains, and pink or orange fungal growth in infected plant tissue [2,3]. One of the main concerns with FHB is the production of mycotoxins contaminating the grain and posing a health risk to humans and animals. *F. graminearum* produces several mycotoxins during barley infection, including deoxynivalenol (DON) [4]. DON is a type of trichothecene mycotoxin that can cause vomiting, diarrhea, and other gastrointestinal problems in animals and humans [5]. The presence of mycotoxins in barley can have significant economic consequences as contaminated grains can be rejected or sold at a lower price due to safety concerns [6]. Therefore, it is essential to implement strategies that limit the risk of FHB and mycotoxins in barley. These may include the use of resistant cultivars, crop rotation, proper planting and harvesting timing, proper storage practices, and the application of chemical fungicides [7]. However, the latter, despite being an indispensable component of antifungal strategies today, poses a threat to the environment and might also induce DON synthesis [4]. Alternative strategies based on environmentally safe strategies should be explored.

Chitosan (CS) is a natural polysaccharide that is derived from chitin, a structural component of the exoskeleton of crustaceans such as shrimp and crabs [8]. It is also a main component of fungal cell walls. CS has been shown to have antifungal activity against a variety of fungal pathogens, including *Fg* [9,10]. CS has been investigated as a potential natural alternative to synthetic fungicides to control *Fusarium* species in agricultural settings. For example, Francesconi, et al. [11] reported antifungal activity of CS against *F. graminearum* and decreased symptoms of FHB of inoculated durum wheat. Buzón-Durán, et al. [12] reported CS activity against *F. culmorum*. Al-Hetar, et al. [13] showed that CS inhibited the growth of *F. oxysporum*, another soil-borne pathogen causing wilt disease in many cultivated plants, including cereal crops, tomatoes, bananas, and cotton. Overall, the antifungal effect of CS against various fungal pathogens, including different species of *Fusarium*, reviewed by Poznanski, Hameed and Orczyk [10], is promising in developing crop protection strategies. However, more research is needed to fully understand the mechanisms of action and optimize the use of CS as a natural fungicide in agricultural settings.

Transcriptomics is a powerful tool that can be used to study plant–pathogen interactions at the molecular level [14]. Using transcriptomics, researchers can identify genes that are differentially expressed in both the plant and the pathogen during infection. This information can help identify key molecular pathways involved in the interaction and can lead to the development of strategies to control the pathogenesis and disease. One approach to transcriptomics is to use high-throughput sequencing technologies, such as RNA-seq, to analyze the transcriptomes of both the plant and the pathogen. RNA sequencing involves isolating RNA from infected tissue and sequencing it to generate millions of short reads that can be aligned to a reference genome or assembled de novo [15]. By comparing the transcriptomes of infected and uninfected tissues, researchers can identify differentially expressed genes and pathways involved in the interaction. Transcriptomics has been used to study plant–pathogen interactions in barley and *Fusarium* pathogens [16]. By identifying the genes and pathways involved in the interaction, researchers can develop new strategies to control plant diseases, such as breeding resistant cultivars or developing new fungicides that target specific molecular pathways. The use of CS is a promising strategy because the compound is environmentally safe, biocompatible, and easily available. It also shows relatively strong antifungal activity, and as reported in this article, inhibits *Fg* growth, and attenuates the expression of toxin-related genes.

The current study aims to investigate the antifungal activity of CS against *Fg* at the molecular level. Testing different batches of CS, we found that only certain types of CS showed strong antifungal activity in vitro, reduced toxin accumulation, and disease symptoms in *Fusarium* inoculated barley leaves. Using in planta pathosystem (barley leaves infected with the *Fg* isolate BW5), we analyzed the fungal transcriptome after CS treatment. This revealed a set of differentially expressed genes (DEGs) and pathways involved in response to the application of CS. For example, genes involved in carbohydrate metabolism and fatty acid synthesis were significantly negatively regulated, while genes involved in stress response and defense mechanisms were found to be up-regulated. Toxin levels in infected leaves were significantly reduced, and the results are discussed in relation to *Fg* transcriptome analysis. By understanding these mechanisms, researchers can develop new strategies for controlling plant diseases and improving crop yield and quality.

## 2. Results and Discussion

### 2.1. Chitosan Antifungal Activity and Minimum Inhibitory Concentration (MIC)

**Figure 1:**
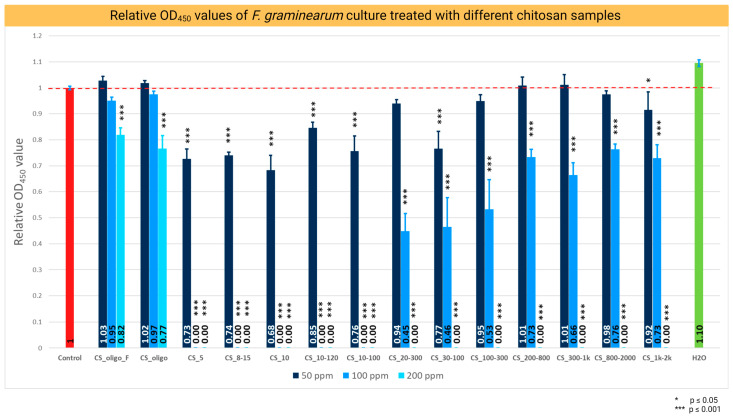
Relative OD_450_ values of *F. graminearum* culture in PDB medium containing 0.01% acetic acid (control) and chitosan at concentrations of 50, 100, and 200 ppm. The OD_450_ readings were taken 120 h post inoculation. The red bar represents relative OD_450_ values of *F. graminearum* in the control medium (PDA with 0.04% acetic acid), medium values, and SD of the relative growth, and the significant *p*-values: *** *p* ≤ 0.001, * *p* ≤ 0.05.

The antifungal effect of different batches of CS was evaluated by measuring the optical density (OD_450_) of *Fg* after 120 h of PDA culture supplemented with 50, 100, and 200 ppm concentrations of tested CS batches in microtiter plate assay (Table 1, Figure 1).

Of the whole collection of CS samples, the batches CS_5, CS_8-15, CS_10, CS_10-120, and CS_10-100 stopped the growth of *Fg* at a concentration of 100 ppm, indicating the MIC value of 100 ppm and the strongest antifungal activity compared with the other samples (Figure 1). The relative value of OD_450_ in the presence of CS_10 was 0.68 showing the strongest inhibition of *Fg* growth in this assay (Figure 1). Batches of medium to high MW 10–120, 10–100, 20–300, 30–100, 100–300, 200–800, 300–1k, 800–2000, and 1k–2k showed descending antifungal activities, clearly lower compared to CS-10. The MIC value for this set of CS samples was 200 ppm. The batches of low MW CS_oligo_F and CS_oligo showed very weak antifungal properties. Low growth inhibition was detectable only at a concentration of 200 ppm (Figure 1).

**Figure 2:**
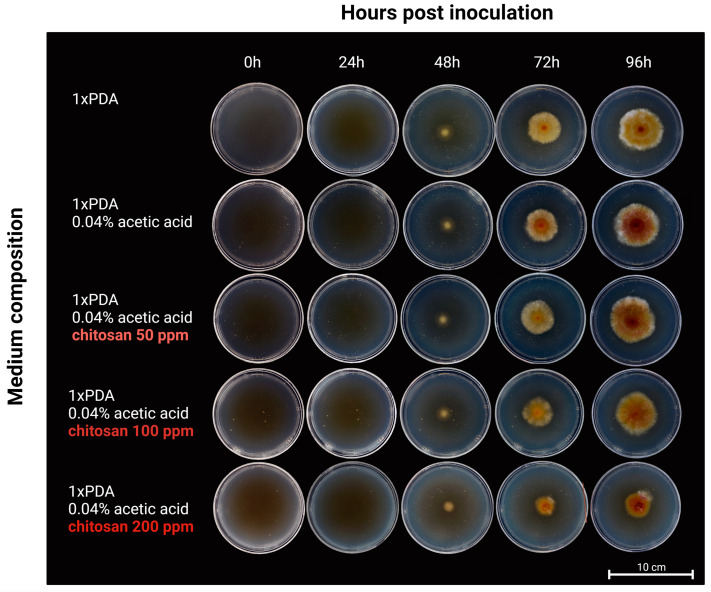
Antifungal activity of the chitosan batch CS_10 shown on PDA Petri plates. Representative pictures, taken at 0, 24, 48, 72, and 96 hpi of Petri plate culture of *F. graminearum* indicated different radii of mycelium on the control (PDA with 0.04% acetic acid) and in the PDA medium containing chitosan.

CS of different providers varied greatly in terms of molecular weight (MW), degree of polymerization (DP), and viscosity of the standardized water solutions. The results showed clear differences in the antifungal properties of the CS batches tested, indicating a significant impact of the physiochemical characteristics of CS on its biological properties (critically reviewed in our recent article describing the characteristics of antifungal activity of CS [10]). The antifungal activity of CS_10 was further confirmed by the Petri plate assay with *Fg* on PDA. The relative area of the mycelium grown on PDA with 50, 100, and 200 ppm of CS_10 was evaluated at 96 h of culture (Figure 2). Growth inhibition was significantly different for the three concentrations tested during 96 h of *Fusarium* culture. The 200 ppm concentrations of CS_10 showed an antifungal impact up to 96 h (Figure 2). Based on the results of the microtiter and Petri plates assays, CS_10 was selected for all other studies and used at a concentration of 200 ppm. This batch of chitosan was characterized by the optimal combination of physicochemical parameters for antifungal activity.

These results are in line with several previous studies reporting the antifungal properties of CS. Park et al. [17] used batches of CS of varying MW (1, 3, 5, and 10 kDa) against nine species of fungi, and they found that the MIC of CS 5 and 10 kDa did not exceed 0.04 mg/mL for all tested species; thus, the two batches showed stronger antifungal activity than the two remaining batches (1 and 3 kDa). El-Mohamedya et al. [18] showed the antifungal impact of high and low molecular weight CS against different species of pathogenic fungi. They reported that low molecular weight CS applied at 0.125% to 1.0% (1250 to 10,000 ppm) inhibited the growth of *Fusarium* species from 25.2 to 100%. However, 10,000 ppm represents a very high concentration that is of no practical use in the field.

### 2.2. Identifying the Chitosan Application Time-Point for Transcriptomics

**Figure 3:**
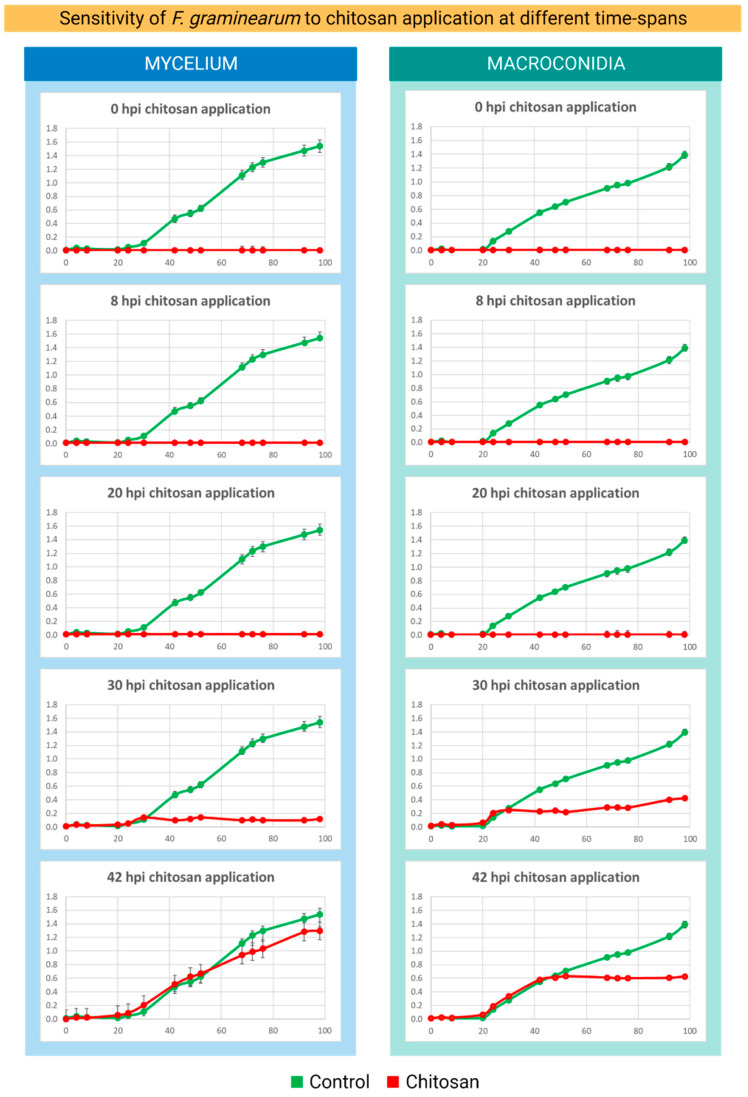
Relative growth of *F. graminearum* in PDB medium (green) and in the PDB medium supplemented with chitosan CS_10 at 200 ppm final concentration (red) at the following time points: 0, 8, 20, 30, and 42 h post inoculation (hpi). The final OD_450_ values were measured at 98 hpi. The green lines represent the growth of *Fg* in the control PDB medium. The red lines represent the growth of *Fg* in the PDB medium supplemented with CS_10.

Transcriptomic is the molecular snapshot of a living cell. The identification of a critical time point is important to detect the transcriptomic of the host–pathogen interaction as well as the pathogen response triggered by CS treatment. Here, we observed from the microtiter plate assays that CS_10 (final concentration 200 ppm) applied 0, 8, and 20 h after PDB inoculation with macroconidia or mycelium inhibited the growth of *Fg* in the culture. The growth of *Fg* was strongly inhibited when the PDB culture was supplemented with CS_10 30 hpi. However, at this point certain *Fg* growth remained as compared to the earlier ones (0, 8, and 20 hpi). Chitosan application at 42 hpi allowed for *Fg* growth, although at a detectably lower rate than the control (Figure 3). This experiment was aimed at finding the critical time points for transcriptomics studies. We reasoned that there would be enough *Fg* cells for transcriptomics when chitosan was applied at 0, 8, or 20 hpi. *Fusarium* growth was also strongly restricted when CS_10 was applied at 30 hpi. The time point of 42 hpi would allow us to analyze the still living *Fusarium* response to CS treatment. In the subsequent transcriptomic experiments conducted on the *Fg*-barley leaf system, CS_10 was applied at 48 hpi on leaves with *Fg* macroconidia. 

Lopez-Moya, et al. [19] conducted a similar time course experiment, where *Neurospora crassa* was subjected to CS applications. The authors observed an inhibitory effect of CS on the growth of *N. crassa* 8 h after germination, which is considered a critical time point for the formation of germ tubes during the growth of conidia. In their study, the application of CS at 0.5 ug/mL reduced conidial germination of *N. crassa* by up to 50% compared to the control. Microscopic tracking of *Fg* during maize infections revealed interesting data on its infection patterns and entry into host cells [20]. The authors observed that *Fg* starts intercellular growth during the initial phase (12–18 hpi) and then began penetration into host cells with intracellular growth patterns (20 hpi onwards). During this initial phase of growth, the fungal hyphae penetrated the plant cell wall and invaded host cells causing brown lesions that were detectable at 72 hpi [20]. Here, in our work, we did not observe *Fg* growth during the initial time points (0–18 hpi), and this could be due to the direct impact of CS_10 on cell membranes and cell processes. After 30 hpi, some growth was observed in treatments, possibly because fungi hyphae escaped CS contact. However, after 42 hpi, the growth of *Fg* treated with CS was significantly lower than the control. 

Furthermore, to confirm the inhibitory effects of CS on *Fg* macroconidia, we performed Trypan blue staining of the cells in liquid culture. This confirmed that CS completely killed the macroconidia as they were all stained blue (Supplementary Figure S1B). Living cells were not stained as shown in the negative control (Supplementary Figure S1A). A mixture of CS_10 treated and CS_10 nontreated macroconidia confirmed that Trypan blue staining discriminated dead from living macroconidia (Supplementary Figure S1C). The sensitivity of germinating conidia to this treatment is important for understanding the impact of CS on *Fg* growth.

### 2.3. Chitosan Treatment Reduces the Accumulation of Mycotoxins of F. graminearum in Barley Leaves

**Figure 4:**
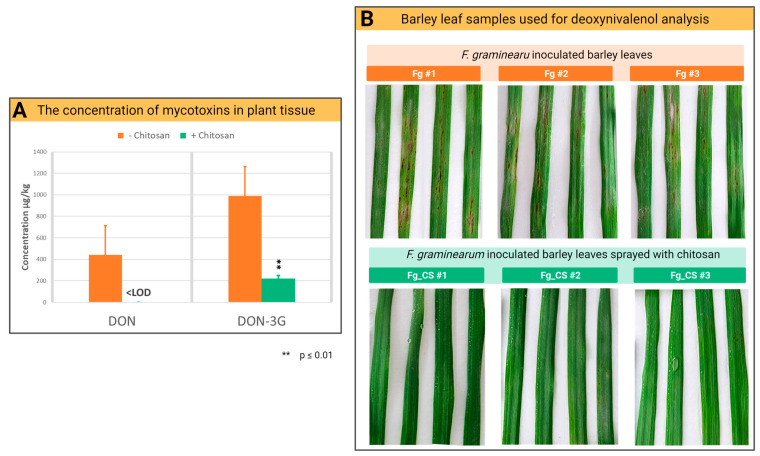
Effect of chitosan on *F. graminearum* toxin production and infection symptoms in barley leaves: (**A**) The application of CS_10 at a concentration of 200 ppm significantly reduced the concentration of toxins (DON and DON-3G) in barley leaves measured at 72 hpi. (**B**) Necrosis symptoms in the positive control (*Fg*-infected barley leaves) and no/fewer symptoms in the *Fg*-infected barley leaves treated with CS_10 show the strong antifungal activity of CS. The photos were taken at 72 hpi.

Deoxynivalenol (DON), a *Fusarium* mycotoxin accumulated in infected crops, poses serious health risks to humans and animals and should be reduced to a minimum in the food chain. To evaluate the antifungal impact of CS on *Fg* mycotoxin production, the solution of CS_10 was sprayed on barley leaves inoculated with *Fg,* and the concentration of DON was analyzed. At 72 hpi in barley leaves with the *Fg* isolate BW-5, a significant level of DON and DON-3G was detected in control plants (Figure 4). The application of CS_10 at a concentration of 200 ppm reduced the level of DON and DON-3G in the samples (Figure 4A) and was associated with a reduction in disease (Figure 4B). In the control leaves, the mean concentration of DON was 440.3 µg/kg in the leaf samples, which corresponded to the high level of accumulation of mycotoxins. In the sample treated with CS_10, the concentration was below the limit of detection (LOD = 150 µg/kg of dry leaf weight) and it was considered 0. The average concentration of DON-3G in the control samples without treatment was 988.9 µg/kg, while in the samples treated with CS_10, the concentration was 222.2 µg/kg. The results indicate a significant reduction, more than 83%, in the toxin level in leaves (Figure 4B).

The estimated necrotic area of barley leaves shows a great reduction in infection symptoms in *Fg*-inoculated and CS-treated leaves compared to control *Fg*-inoculated leaves (Figure 4B). Consistent with the above is the significant, more than eight times lower relative transcript reads of *Fg* housekeeping genes found in the transcriptomic data (Supplementary Table S2 and Figure S3). Both sets of results, i.e., reduced necrotic area and reduced transcript reads of *Fg* housekeeping genes, indicate strong inhibition of *Fg* growth and a lower number of fungal cells in infected barley leaves. 

CS has been shown to have antifungal properties and has been used in various combinations to retard fungal growth and toxin synthesis. Wu, Wan, Lu, Wang, Zhong, Schwarz, Chen and Rao [9] used electrostatically stabilized CS with lecithin emulsion and reported antifungal activity in terms of DON synthesis and growth inhibition. They showed that rice cultures inoculated with *Fg* accumulated up to 98.6% less toxin when treated with high molecular weight CS. The authors described that various physiochemical parameters of CS affected its antifungal properties. The strongest inhibition of DON accumulation showed high molecular weight CS, which was formulated with lecithin and was stronger than medium and low molecular weight CS [9]. Deshaies, et al. [21] reported that spray with CS (195 kDa, 10–50 cps viscosity and 90% DD) alleviated Fusarium head blight (FHB) symptoms in wheat grown in greenhouses by inhibiting spore germination and hyphal growth. The metabolomic study indicated changes in various pathways, including the JA-dependent resistance pathway [21].

### 2.4. Fg Transcriptomics in Planta and Chitosan Applications

#### 2.4.1. General Characteristics of *F. graminearum* Transcriptomes (RNA-Seq)

**Figure 5:**
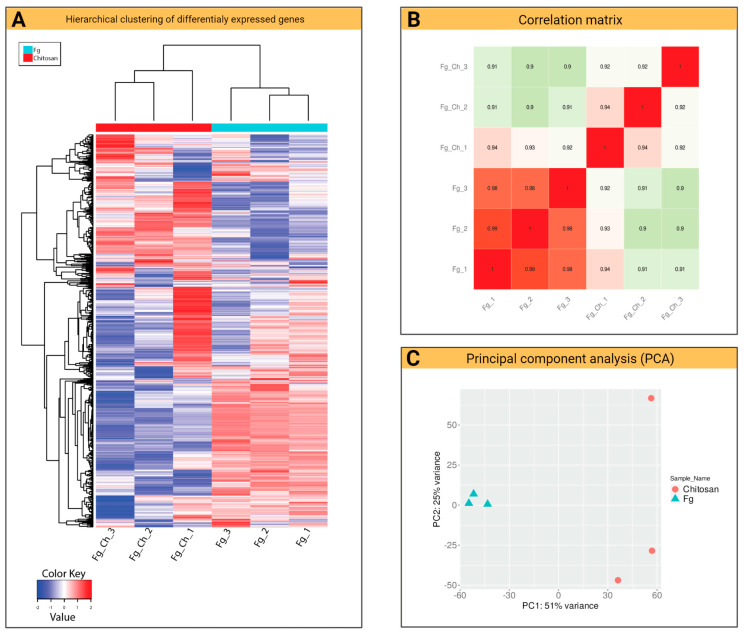
Response of *F. graminearum* to chitosan CS_10 at 200 ppm treatment: (**A**) Hierarchical clustering of DEGs that respond to *Fg* against treatment with CS_10 at 48 hpi. The red color shows induced genes, whereas the blue color shows repressed genes. (**B**) Hierarchical clustering based on Pearson correlation coefficients during experimental conditions. (**C**) The PCA of the transcriptome of six samples. Principal component 1 (PC1) describes the variance (51%) between biological replicates of *Fg* samples treated with CS_10 at 48 hpi. The principal component 2 (PC2) describes the variance (25%) between the biological replicates of the control samples of *Fg*.

The *Fg* samples (control) clustered in the different areas of the plot and the treatments showed some variability in one sample of the individual behavior of *Fg* in response to CS_10 treatment. The hierarchical clustering of *Fg* DEGs against the CS_10 treatment showed a repressed gene pattern compared to the control genes (Figure 5A). The blue color shows the repression, while the red color represents the induced genes. 

The heat map illustrates a general comparison of the transcriptomes of both organisms in response to specific stress. The Pearson correlation matrix revealed a linear relationship between the samples during the experimental conditions and grouped them. The range of the values of the control samples (1.0–0.98) and the samples treated with CS_10 (0.91–0.94) showed a positive correlation between the experimental groups (Figure 5B). ‘The Pearson correlation measures the strength of the linear relationship between two variables. It has a value between −1 and 1, with a value of −1 meaning a total negative linear correlation, 0 meaning no correlation, and + 1 meaning a total positive correlation [22]’.

A significant response of DEGs was observed when *Fg* was treated with the CS_10. An overall decrease in *Fg* gene expression was observed with a log2 fold change of >2.0 (*p*-value < 0.05) compared to the control (Figure 5). The principal component analysis (PCA) of the aligned transcripts showed similarity in the biological replicates of the experimental unit. The analysis is widely used to compare variables simultaneously and to explore the variability among biological replicates. The PCA plot revealed the variance scores of the first (PC1) and second (PC2) of 51% and 25% among the six samples (Figure 5C).

#### 2.4.2. Volcano Plot and *k*-Means Clustering

**Figure 6:**
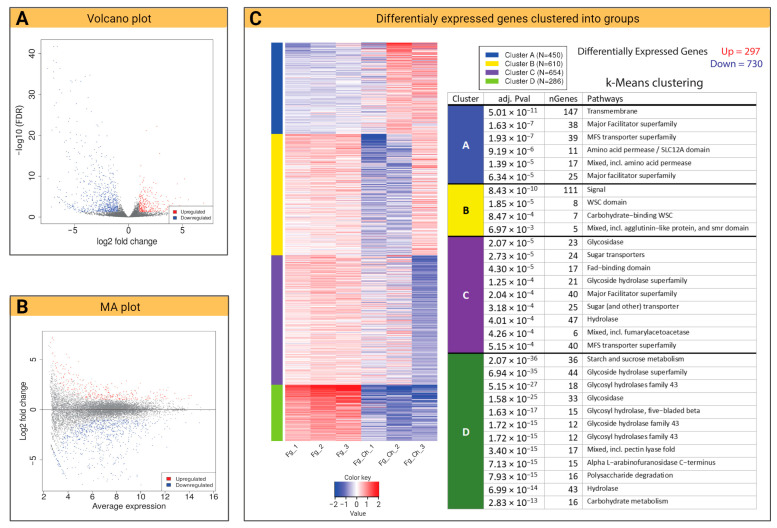
Overview of the transcriptome of *F. graminearum* after chitosan CS_10 treatment: (**A**) Volcano plot exhibiting default log2 fold change against adjusted *p*-value (0.05). (**B**) MA plot generated against log2 fold change ranging from −1 to 1 default threshold level. (**C**) *K*-means enrichment analysis clustering group of genes into four different groups (A, B, C, D) based on their core molecular and biological functions. Blue/red colors indicate down- or up-regulation respectively.

The dispersion patterns of the DEGs visualized through the volcano plot and the MA plot revealed a statistical difference between the two treatments. Volcano plots typically use the negative fold change of base-10 and base-2 log fold change, respectively, to show the statistical significance of the difference of the size for each gene in the comparison [23]. The wide dispersion of down-regulated genes on the *y*-axis demonstrates the lower *p*-values and thus, more log2 fold change (Figure 6A). The dispersion of the values on the *x*-axis represents the variability of the level of expressions, and the dots near the eruption point (0) represent identical mean expression levels (Figure 6B). In the MA plot representation, the log2 fold changes (*y*-axis) are expressed as dispersions versus normalized mean counts plotted on the *x*-axis (Figure 6B). The data points in the MA plot coincide with the expression pattern observed in the volcano plot with a significant number of down-regulated genes along the *y*-axis (-ve values below 0) (Figure 6B). Furthermore, we analyzed the expression pattern in the samples based on the clustering of *k*-means. The model was selected to group a maximum of the top 2000 DEGs of each biological sample and group them into four clusters based on the promoter enrichment analysis [24]. From the results generated, we observed a significantly higher proportion of down-regulated genes (730) as compared to up-regulated genes (297) (Figure 6C). A dense cluster of down-regulated genes was found to be involved in the glycolysis and starch metabolic pathways. However, the transmembrane movement of molecules such as amino acid permeases and genes from the MFS transporter family was up-regulated after CS_10 treatment. 

#### 2.4.3. Chitosan Impact on DON-Related Transcripts of *F. graminearum*

Here, we observed a significant reduction in DON accumulation in barley treated with CS_10 *in planta* designed experiments (Figure 4). The enrichment analysis of DEGs showed several different gene groups that were dominantly involved in biological processes (BP), molecular functions (MF), and cellular components (CC). Table 2 shows an overview of the top four major genes involved in these pathways/processes that are up- and down-regulated. RNA-Seq data revealed a significant down-regulation of *Fg* genes; a total of 90 genes were involved in the carbohydrate metabolic process, 17 in the catabolic process of polysaccharides, and 316 in catalytic activities (Table 2).

The time point for the *Fg* transcriptome was established as 72 hpi and 48 h after treatment with batch CS_10 chitosan. To find the molecular regulation behind this, we analyzed transcripts of genes that function in the trichothecene biosynthesis pathway. Over the past decade, a significant part of the research was conducted on the routes of trichothecene biosynthesis in the *Fg* and related species [4,25]. The *Fg* genome encodes 16 trichothecene biosynthesis genes (*TRI* genes), including *TRI1*, *TRI3-TRI16*, and *TRI101* that reside in a genomic region of 25 kb [26]. Wang, et al. [27] identified the trichothecene production capacity of different strains of *Fg* isolates and reported DEGs associated with variable trichothecene accumulations between different fungi. This group identified some new candidate genes (FGSG_01403, FGSG_10571, FGSG_01959, FGSG_09786, FGSG_03796, FGSG_02367, FGSG_07765, FGSG_01786, FGSG_02113, FGSG_02366, FGSG_02371) that are associated with trichothecene biosynthesis in *Fg* species. However, as Wang, et al. [28] stated, this needs further characterization for molecular and biological functions. For example, proteome analysis of the FGSG_01403 protein showed negative regulation with trichothecene production. Knockout mutations in this gene resulted in increased production of trichothecenes in the *Fg* isolate PH1 [27]. 

**Figure 7:**
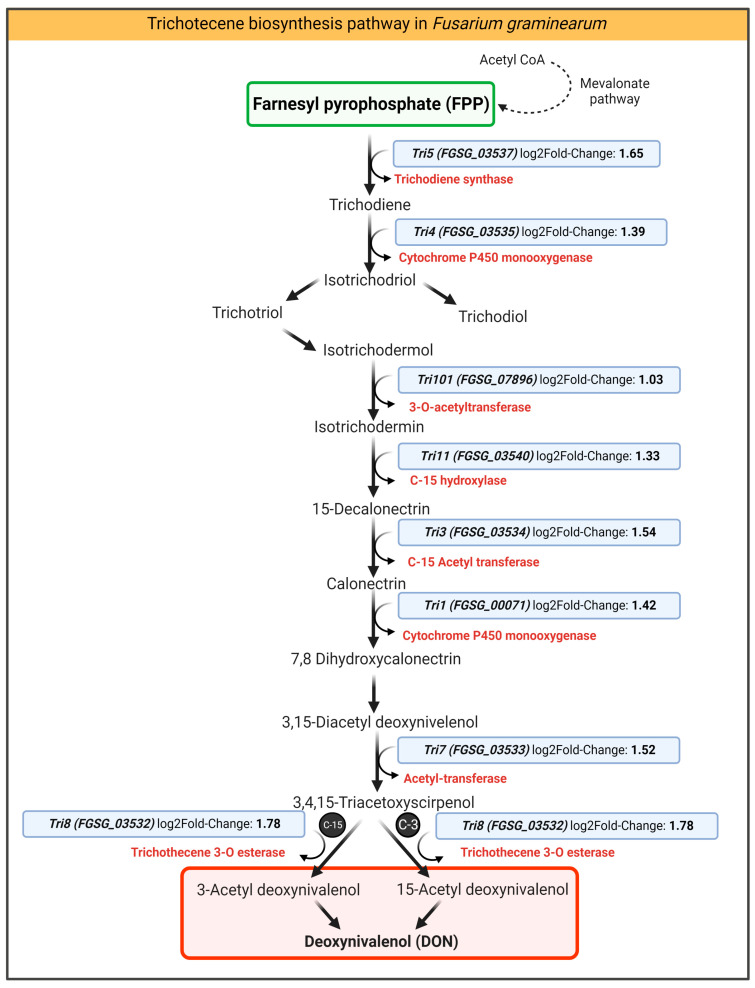
Schematic illustration of the DON biosynthesis pathway in *F. graminearum.* The table represents the values of DEGs related to DON biosynthesis observed in this study. The CS_10 applications affected the *Fg* trichothecene pathways.

Here, in our data, we observed up-regulation of genes from the trichothecene biosynthesis pathway: *Tri5*, FGSG_03537, 3.13-fold change; *Tri4*, FGSG_03535, 2.62-fold change; *Tri101*, FGSG_07896, 2.04-fold change; *Tri11*, FGSG_03540, 2.46-fold change; *Tri3*, FGSG_03534, 2.91-fold change; *Tri1*, FGSG_00071, 2.68-fold change; *Tri7*, FGSG_03533, 2.87-fold change and *Tri8*, FGSG_03532, 3.44-fold change (Figure 7, Supplementary Table S2).

Up-regulation of *TRI* genes (Figure 7, Supplementary Table S2) and significantly lower concentration of DON and DON-3G in CS treated leaves (Figure 4) could be a cumulative result of inhibition of *Fg* growth and a lower number of fungal cells in infected barley leaves (Section 3.3, Supplementary Figure S3) and significant down-regulation of genes of general metabolism that are associated with toxin synthesis. As reviewed by Chen, Kistler and Ma [4], trichothecenes biosynthesis is strictly associated with glycolysis because one of the glycolysis by-products, farnesyl pyrophosphate (FPP), is a substrate for DON biosynthesis [29]. 

Here, we observed a strong and significant down-regulation of several key genes involved in glycolysis: FGSG_00571 (0.005-fold change), FGSG_03049 (0.019-fold change), FGSG_03628 (0.011-fold change), FGSG_03795 (0.008-fold change), FGSG_06445 (0.010-fold change), FGSG_07695 (0.008-fold change), FGSG_08011 (0.010-fold change), FGSG_10999 (0.10-fold change), FGSG_11208 (0.016-fold change), FGSG_11258 (0.007-fold change), FGSG_11428 (0.018-fold change), FGSG_12551 (0.012-fold change), FGSG_07694 (not assigned) and FGSG_13855 (50.825-fold change) and metabolic processes: FGSG_03624 (0.179-fold change), and FGSG_10999 (0.010-fold change) (Supplementary Table S2). 

#### 2.4.4. DEG and KEGG Path Analysis

**Figure 8:**
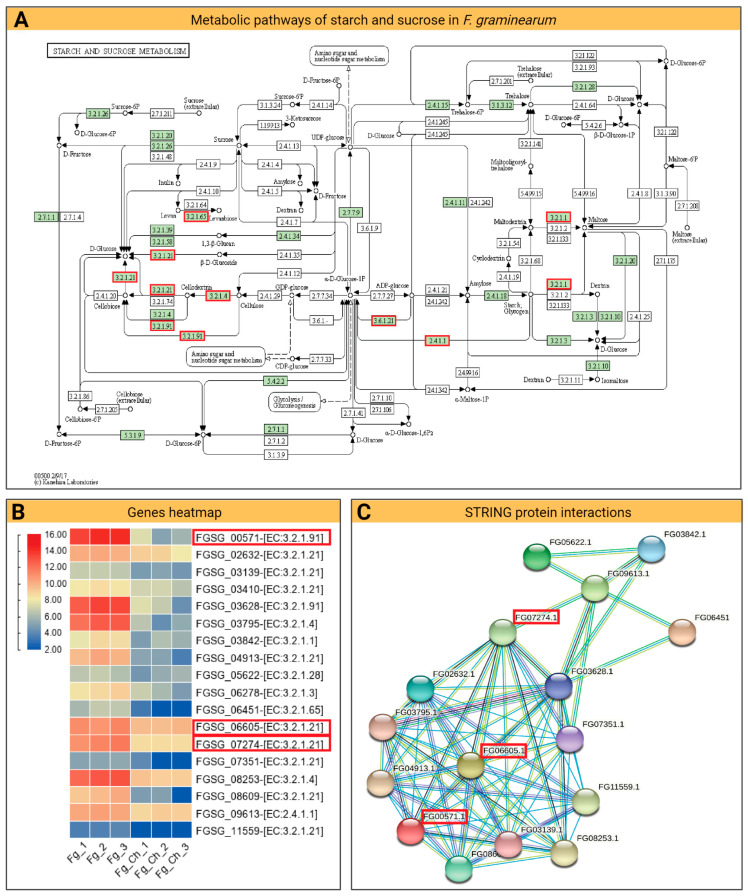
Metabolic pathways of starch and sucrose in *F. graminearum* and their relationship to the transcriptome: (**A**) The KEGG pathway illustration of the starch and sucrose metabolic process in *Fg* cells. Reprinted/adapted with permission from Refs. [32,33,34], 2023, KEGG, Kanehisa Laboratories. The red rectangles correspond to genes down-regulated after CS_10 treatment. (**B**) Heatmap of important *Fg* genes down-regulated with CS_10 treatment. Red corresponds to higher gene expression, while blue corresponds to lower gene expression. (**C**) Diagram of the protein–protein network showing the interaction among different genes. The red rectangles describe the significant genes.

Based on the fold-change values obtained by DESeq2 for all genes, pathway analyses were performed to look for DEGs specifically involved in a crucial biological process. For this purpose, the diagram of the KEGG pathway [30] was drawn and we looked for the significantly down-regulated genes involved in that specific pathway (Figure 8). 

For top-down-regulated genes in *Fg* (ensemble IDs; FGSG_00571, FGSG_06605, and FGSG_07274), significant involvement was observed at various stages of starch and sucrose metabolism. Here, in the KEGG diagram, FGSG_00571 was involved in the binding of cellulose and cellodextrin and conversions to D-glucose. The CS_10 application strongly reduced the expression of this gene (0.005-fold change) compared to the control (Supplementary Table S2). Similarly, the other genes, FGSG_06605 and FGSG_07274, have a significant down-regulation of 0.56- and 0.141-fold change, respectively (Supplementary Table S2). Both genes play a key role in the carbohydrate metabolic process in fungi cells that function in the conversions of β-D-glucose, cellodextrin, cellobiose into D-glucose, and ADO-glucose into α-D-glucose-IP. Down-regulation of the key pathways may have a strong impact on carbohydrate metabolism and may lead to *Fg* cell death, as observed in the current experiment. To see the predicted coordination among these genes, the STRING database [31] was used to draw protein–protein interactions that included direct (physical) and indirect (functional) associations. The outcome scheme showed a strong network of interaction among FGSG_00571 and FGSG_06605 that cointegrated strongly with several other proteins in the network system (Figure 8). 

#### 2.4.5. Fatty Acid Degradation Pathway

**Figure 9:**
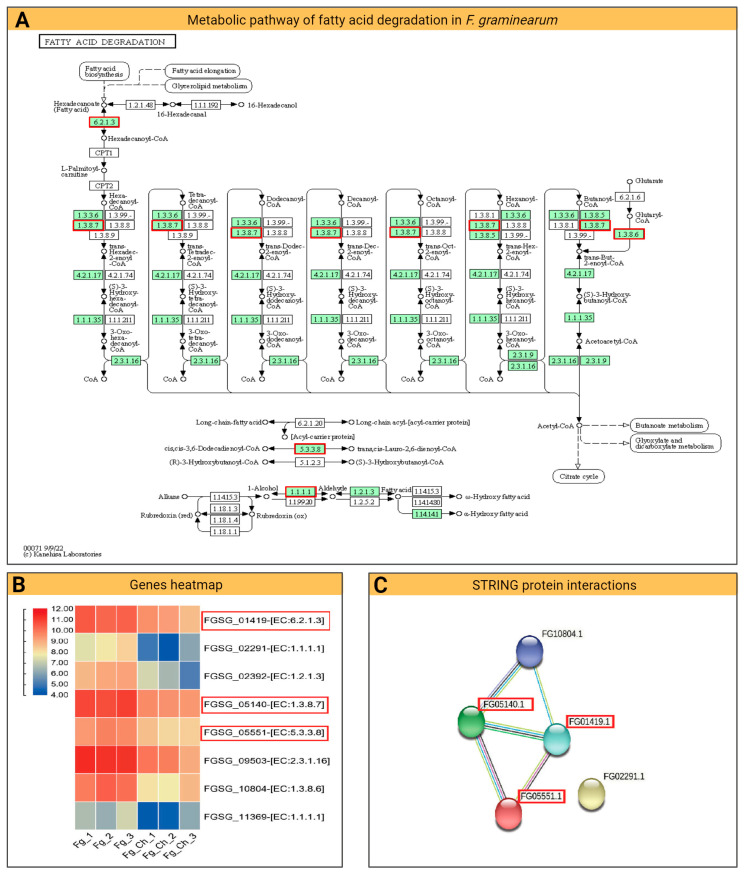
Fatty acid metabolic pathways in *F. graminearum* and their relation to the transcriptome: (**A**) The KEGG pathway illustration of the fatty acid metabolic process in *Fg* cells. Reprinted/adapted with permission from Refs. [32,33,34], 2023, Kanehisa Laboratories. The red rectangles correspond to genes down-regulated after CS_10 treatment. (**B**) Heatmap of important *Fg* genes down-regulated with CS_10 treatment. Red corresponds to higher gene expression, while blue corresponds to lower gene expression. (**C**) Diagram of the protein–protein network showing the interaction among different genes. The red rectangles describe the significant genes.

Fatty acid metabolism is important for the β-oxidation of fatty acids, the release of secondary metabolites, and the catalytic degradation of reactive oxygen species (ROS). Fatty acid pathways are involved in *Fg* pathogenicity [35], colonization in different hosts [36], maintaining cell wall integrity [37], and vegetative and reproductive growth of fungi [38]. Here, in this study, CS_10 used at 200 ppm significantly affected the *Fg* transcriptome and down-regulated several genes that are primarily involved in the metabolic pathways of fatty acids (Figure 9). The key genes found in multiple cores of the fatty acid pathways are FGSG_05140, FGSG_05551, and FGSG_01419. The FGSG_01419 gene is involved in the conversion of hexadecanoyl-CoA to hexadecanoate during fatty acid degradation. The paralogue of this gene in *F. asiaticum* revealed a role in virulence and vegetative growth when observed through mutation studies. More specifically, deletion mutants of the two septin genes (*FaCdc3* and *FaCdc12*) in wheat-infecting *F. asiaticum* showed reduced levels of DON in wheat kernels, restricted mycelial growth, and irregular hyphae [39]. Here, the CS_10 application reduced the expression of this gene (FGSG_01419) by up to 0.433 times (Supplementary Table S2), which could reduce the metabolism of fatty acids. Similarly, another gene, FGSG_05140, down-regulated by 0.386 times (Supplementary Table S2), was found to function in several stages of fatty acid degradation pathways. The *Fg* FGSG_05551 is another critical gene that has catalytic activity in the conversion of long-chain fatty acids into short-chain fatty acids. Here, a down-regulation of 0.436 (Supplementary Table S2) was observed after treatment with CS_10.

#### 2.4.6. Chitosan Treatment Up-Regulates Stress-Responsive Genes of *F. graminearum*

CS_10 triggered a transcriptome response in *Fg* and was measured in terms of some major molecular functions such as oxidoreductase, transmembrane transport, and mycotoxin production. Significant up-regulation of genes involved in ergosterol biosynthesis and cell wall synthesis is a type of antistress strategy adopted by *Fg*. For example, FGSG_11656 is involved in fatty acid synthase activity regulated by S-acetyltransferase activity, enoyl-[acyl-carrier-protein] reductase (NADH) activity, and/or transferase activity. The gene showed a strong up-regulation of 48.370 times after CS_10 treatment (Supplementary Table S2). The synthesis of fatty acids is important for vegetative growth and for the response to stress in eukaryotes. In *Fg*, deletion mutation of the ergosterol pathway gene (elongase gene; *Fg ELO2*) decreased fungi sensitivity to tebuconazole and also reduced virulence [40]. In the case of chitosan-treated *Fg*, we observed a strong up-regulation of genes connected directly with response to oxidative stress such as: *catalase 1* (FGSG_06554), *catalase 3* (FGSG_06733), *catalase 4* (FGSG_12369) up-regulated, respectively, 6.32, 4.02 and 2.18 fold [41].

**Figure 10:**
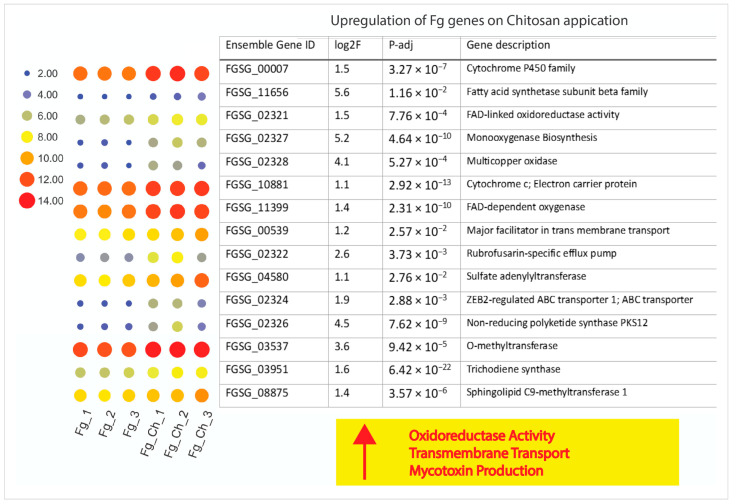
Up-regulation of *F. graminearum* genes after chitosan treatment. Heat map display of up-regulated genes corresponding to the abundance of their transcripts. The bigger the size of the dots, the more copies of that specific gene are expressed.

Superoxide dismutases, as ROS scavengers, are the first line of defense against stressful situations. Here, 17,036- and 36,843-fold up-regulations of the multicopper oxidase (FGSG_02328) and flavin adenine dinucleotide (FAD) protein (FGSG_02327), respectively, were a strong response against chitosan CS_10 treatment (Figure 10, Supplementary Table S2). Up-regulation of superoxide dismutases (Cu-Zn) in *Fg* corresponds to the elimination of ROS and the enhancement of virulence in wheat head infections [42]. The response of *Fg* to oxidative stress is related to the synthesis of mycotoxins [41]. The transcription factor *Fgap1* (FGSG_08800) (1.2-fold change) activates the transcription of antioxidant enzymes while affecting the regulation of *Tri* genes and the production of trichothecenes [41,43].

#### 2.4.7. qPCR Validation of RNA-Seq Data

The expression of selected genes by qPCR verifies the results of the RNA-Seq data (Supplementary Figure S2). To confirm this, we selected four different genes from *Fg* that showed significant down- and up-regulation after treatment with CS_10. The same template RNA used for mRNA sequencing was subjected to qPCR and relative quantification was measured using the 2^−ΔΔCt^ method. The strongest down-regulation was observed in the case of the FGSG_00571 gene (0.005-fold change) and was also consistent with lower expression in the qPCR data. Similarly, the up-regulated expression of FGSG_0007 was consistent with the qPCR data, and the results confirmed the RNA-Seq approach to quantify the expression. Previously, several articles have used qPCR as an alternative to gene expression data to verify the authenticity of results [27,40,44].

## 3. Materials and Methods

### 3.1. Materials

In this study, a set of CS batches, representing various physicochemical parameters (viscosity, molecular weight, degree of deacetylation) and of different origins from different providers were used (Table 1). Acetic acid (99.5–99.9%) was supplied by POCH, Poland. Sodium hydroxide (NaOH) was supplied by POCH, (Warsaw, Poland), and Trypan blue was supplied by Pol-Aura, (Warsaw, Poland). Potato dextrose agar (PDA) and Potato dextrose broth (PDB) were supplied by ROTH, (Karlsruhe, Germany). Milli-Q water was used to prepare dilutions of the CS samples. 

### 3.2. Methods

Different methods were used to evaluate the antifungal effects of chitosan on *Fg* growth. *Fg* toxin production was also analyzed. The following section describes the methods involved in these experiments.

#### 3.2.1. Chitosan Solution Preparation

The stock solution of each CS sample was prepared in the same manner. An amount of 3 g of specified CS was dissolved in 1 L of Milli-Q water containing 1% acetic acid (pH 3.0). The solution was stirred overnight at room temperature. The stock solution was then diluted with Milli-Q water at different concentrations, and the pH of the solution was set at pH 5.6 with NaOH. The solutions were then filtrated (0.22 µm).

#### 3.2.2. Production of Macroconidia of *F. graminearum*

The *F. graminearum* BW5 strain was obtained from the collection of the Plant Breeding and Acclimatization Institute (Radzikow, Poland). For this purpose, *Fg* was cultured on a plate with PDA medium for several days and then inoculated through 3–4 cm^2^ pieces of PDA into a 150 mL autoclaved medium (adapted V8) containing 170 mL/L potato and vegetable juice (Fortuna^®^, Poland) and 1.5 g/L calcium carbonate in a 500 mL flask. The flasks were shaken in a rotating incubator at 120 rpm at 25 °C under UV for 2 weeks. The presence of macroconidia was checked under a light microscope (Nikon^®^, Tokyo, Japan). The mixture was filtered through autoclaved Miracloth. The final macroconidia concentrations were counted under a light microscope (Nikon^®^, Tokyo, Japan) using the Fuchs–Rosenthal counting chamber. Macroconidia were stored at −80 °C.

#### 3.2.3. Determination of the Impact of Chitosan on *F. graminearum* Growth

- *Antifungal activity of various batches of chitosan*

A 96-well microtiter plate assay was used to determine the antifungal activity of each batch of CS. Each well in the plate contained PDB medium supplemented with 50, 100, and 200 ppm of the tested CS sample. The wells were inoculated by adding 10 µL of *Fg* macroconidia suspension (1 × 10^5^ spores/mL). The plates were incubated for 120 h in the dark at 25 °C. At the indicated time points, the optical density (OD_450_) of each well was measured. Relative optical density was counted by subtracting read-outs from the noninoculated wells, which acted as a background. Each measurement was related to control wells containing PDB medium, inoculated with *Fg*, but without the addition of CS.

- *Petri plates assay*

The batch of CS with the strongest antifungal activity CS_10 was further tested to determine the effect of CS on *Fg* surface growth in the PDA culture. To confirm the results of the in vitro microtiter assay in Petri plate assay, 10 μL of macroconidia suspension (1 × 10^5^ spores/mL) was added to the center of the PDA medium supplemented with CS_10 at a concentration of 0 (control), 50, 100, and 200 ppm. Photos were taken after each 24 h interval.

- *Time-dependent response of Fg macroconidia and mycelium to chitosan application*

To investigate the time-dependent response of *Fg* to chitosan application, the PDB *Fg* culture was carried out on two *Fg* developmental stages: macroconidia and mycelium. The PDB culture carried out in a 96-well microplate was inoculated alternatively with 10 µL of *Fg* macroconidia suspension (1 × 10^5^ spores/mL), or with freshly grown *Fg* mycelium transferred by a pipette tip. Each well was supplemented with 200 ppm of CS_10 for 0, 8, 20, 30, and 42 h after inoculation (Figure 3) and the culture was carried out up to 100 h in the dark, at 25 °C. The *Fg* growth was monitored by measuring optical density (OD_450_) of each well.

- *F. graminearum macroconidia Trypan blue viability test*

To further investigate the impact of CS on *Fg* macroconidia, Trypan blue staining was used. The protocol was adopted from Boenisch and Schäfer [45]. For this purpose, PDB medium (100 µL) supplemented with CS_10 (200 ppm) was inoculated with macroconidia suspension (1 × 10^5^ spores/mL). Trypan blue (100 μL) was added and incubated at 25 °C in the dark for 20 min. The whole suspension was mixed by pipping and 10 μL was transferred to a glass slide for examination of *Fg* macroconidia under a white-light microscope (Nikon^®^, Tokyo, Japan).

- *Statistical analysis*

The data presented in the graphs are the mean ± standard error of three replicates of each treatment. The ANOVA test and the least significant difference (LSD) post hoc test were performed using Statistix software (Statistics 8.1, Analytical Software^®^, Tallahassee, FL, USA). Results were designated significant at *p* < 0.05 * and designated with different * symbols.

#### 3.2.4. Inoculating Barley Leaves with *F. graminearum*

Seeds of *Hordeum vulgare* cv. Golden Promise were placed on glass balls inside a Petri plate with the addition of water. The seeds were imbibed for 24 h (4 °C, dark) and 48 h for initial germination (21 °C, dark). The germinated seeds were planted in plastic pots (15 cm × 10 cm) filled with substrate soil (Aura-Hollas^®^, Paslek, Poland). The plants were grown for one week under controlled conditions (20 °C with a 16 h light/8 h dark photoperiod). For fungal inoculum, 1 mL of *Fg* macroconidia suspension (2 × 10^5^ conidia/mL) was added to the Eppendorf tube with 1 mL of 10% PDB to induce germination. BREAK-THRU S301 surfactant (Evonik^®^, Essen, Germany) was added to the final concentration of 0.01%. Two droplets (20 μL each) of this inoculum mix were placed on the distal parts of the barley leaf. The entire plant was covered under a cellophane foil hood to maintain a relatively high humidity inside and placed in the dark for 24 h after inoculation. After 24 hpi, the cellophane hood was removed and barley leaves were sprayed with CS_10 at a concentration of 200 ppm. 

#### 3.2.5. Deoxynivalenol Analysis in Barley Leaf Samples

The pre-weighed barley leaf samples (580–990 mg) were homogenized in 50 mL Falcon tubes in 5 mL of water with the Unidrive homogenizer (CAT Scientific^®^, Paso Robles, CA, USA) for 2 min. The samples were centrifuged (MPV, Med. Instruments^®^, Poland) at 10,730× *g* for 10 min, and 4 mL of collected supernatant was diluted with 2 mL of phosphate-buffered saline (PBS). A total of 3 mL of the mixture was passed through the immunoaffinity column (IAC) adapted for DON isolation (Vicam^®^, Milford, MA, USA) at a rate of 1–2 drops per second. Subsequently, the column was rinsed with 2 mL of PBS and 2 mL of deionized water at a rate of 2–3 drops per second. The analytes were eluted from the column first with 0.5 mL of methanol and then with 1.5 mL of acetonitrile. The eluate was collected in a reaction bowl and the solvent was evaporated under a stream of nitrogen. The samples were redissolved in 10% acetonitrile solution and the extract was filtered through a 0.45 µm nylon syringe filter. Each test sample was prepared in two independent replicates. Each independent replicate (on the same sample extract) was prepared twice.

The analytes were analyzed using the technique of high-performance liquid chromatography coupled with a UV detector (A Knauer^®^ K 1001 HPLC instrument, Berlin, Germany). The analyses were separated on a Cosmosil 5C18-AR-II, 4.6 mm × 250 mm chromatography column (Nacalai Tesque, Kyoto, Japan) thermostatic at 45 °C. Analytes were separated in the isocratic mode. The mobile phase was an aqueous solution of acetonitrile (10%) with a flow rate of 1 mL/min. The analytical wavelength set on the UV detector was 218 nm. To determine the concentrations of the tested analytes in the samples, seven-point calibration curves were prepared. These curves were created as a result of the analysis of a mixture of standard solutions prepared by successive dilutions of 0.05 and 1.60 µg/kg of DON and DON-3G, respectively. The calibration curves were characterized by high values (>0.99) of the R2 coefficients. The limits of detection (LOD) and limits of quantitation (LOQ) values were determined on the basis of signal-to-noise (S/N) analysis. For LOD and LOQ, these were the analyte concentration values at which the S/N was 3 and 10, respectively. LOD values for DON and DON-3G were 150 and 180 µg/kg, respectively, while LOQ values were 500 and 600 µg/kg for DON and DON-3G, respectively. Recovery (R%) and repeatability of the method (expressed as relative standard deviation, RSD%) were also determined for fortified barley leaf samples (600–2400 µg/kg). Depending on the level of fortification and the analyte analyzed, the recovery value of the method ranged was from 91 to 109%, while repeatability did not exceed 14%.

### 3.3. RNA-Seq Data Analysis

#### 3.3.1. RNA Extraction and mRNA Sequencing

Total RNA was extracted from 100 mg of barley leaves infected with *Fg* using the RNA isolation kit (Zymo-R2072, Irvine, CA, USA), RNA extraction buffer (50 mM Tris-HCl pH = 8.0, 150 mM LiCl, 5 mM EDTA pH = 8.0, 1% SDS), Trizol reagent, and phenol: chloroform (1:1), 80% ethanol) according to the manufacturer’s protocol. The isolated RNA was quantified using a NanoDrop spectrophotometer (NanoDrop Technologies^®^, Wilmington, DE, USA). Quality was assessed using 1% agarose gel and running on BioAnalyzer 2100 (Agilent Technologies^®^, Santa Clara, CA, USA). Samples having 260/A280 > 2.0, RIN > 8, and concentration >50 ng/µL were selected for downstream applications. Approximately 2 μg of high-quality RNA was sent to a biotech company (GENEWIZ^®^, Leipzig, Germany) to prepare the Illumina standard RNA library with polyA selection. The company performed Illumina NovaSeq sequencing with an estimated data output of ~20 M paired-end reads with a quality score of Q30. The company provided FASTQ format data that were used for further analysis.

#### 3.3.2. The Quality Control and Mapping Sequence Read

The FastQ raw data were processed for quality control checks using the FastQC toolkit v0.12.0 (https://www.bioinformatics.babraham.ac.uk/projects/fastqc/, accessed on 14 December 2022) with standard command lines. High quality reads with sufficient base length (Phred Score > 30) were retained, while all the low quality reads and adapter sequences were subsequently trimmed using Trimmomatic program (http://www.usadellab.org/cms/?page=trimmomatic, accessed on 17 December 2022) [46]. The genome of *Fg* (Assembly: GCA_000240135.3) and the gene annotation file (GFF3) was retrieved from EnsemblFungi (https://fungi.ensembl.org/index.html, accessed on 17 December 2022). The trimmed reads were aligned to the reference genome using Bowtie v2.5.0 [47] with default settings. The output alignment (.sam) was converted to a .bam file using SAMtools. The transcript reads were counted using featureCounts [48], and the differential expression analysis was performed using DESeq2 (Bioconductor R package) [49]. Genes with false discovery rate (FDR, adjusted *p*-value) < 0.05 and log2 fold change > 2 between two experimental conditions were designated as differentially expressed genes (DEG).

#### 3.3.3. Analyzing RNA-Seq Outputs

To determine the variations between treatment samples, a two-dimensional principal component analysis (PCA) was performed using the Bioconductor R PGSEA package (https://www.bioconductor.org/packages//2.10/bioc/html/PGSEA.html, accessed on 20 December 2022). The percentage variance was detected with the default settings that depend on the adjusted *p*-values to rank the specific gene set pathways according to biological processes. For functional enrichment analysis (Gene ontology (GO), and Kyoto Encyclopedia of Genes and Genomes (KEGG)), the iDEP server was used to interpret the data with an FDR cutoff < 0.05 [50]. GO data were used to find gene groups involved in specific biological processes (BP), molecular functions (MF), and cellular components (CC) with a significant *p*-value of 0.05 and log2 fold change > 2.0. Other expression patterns such as *k*-means clustering were performed for the top 2000 DEGs to look for their participation in different processes across all samples [50]. Other general features such as heatmaps, volcano plots, and MA plots were also constructed using the iDEP web portal (http://bioinformatics.sdstate.edu/idep96/, accessed on 20 December 2022) to obtain meaningful results. To detect the DEG expression networks, we used the WGCNA package in R through the iDEP network analysis tool. This package utilizes the normalized gene expression data to find co-expression networks which can be visualized in Cytoscape [51]. Protein–protein interaction networks were drawn by using the top DEGs in the selective pathway in the STRING server (https://string-db.org/, accessed on 21 December 2022). The targeted organism was *Fusarium graminearum* and multiple protein interactions were identified with the following parameters: Network Type; full STRING network; required score; medium confidence (0.400) and FDR stringency; medium (5%).

### 3.4. Quantitative Real-Time PCR (qRT-PCR) of Selected F. graminearum Genes

Total RNA extracted (Section 3.3.1) was treated with 2 U of RiboLock RNase inhibitor and 2 U of DNase1 (Roche Diagnostics^®^, Munich, Germany) to remove genomic DNA impurities. To confirm the absence of DNA traces, 100 ng of DNase-treated RNA was subjected to a polymerase chain reaction (PCR) with *Fg*-Elongation Factor 1 (FGSG_08811; [52]) gene primers (Supplementary Table S1) for possible genomic amplification. The lack of any template product after 37 cycles of amplification confirmed the purity of DNA-free template RNA. Then, 2 µg of this RNA was subjected to cDNA synthesis using the RevertAid First Strand cDNA Synthesis Kit (ThermoFisher Scientific^®^, Waltham, MA, USA) following the manufacturer’s instructions. A 50:50 ratio of Oligo-dt and random hexamer primers was used to synthesize the maximum length of the cDNA templates. The resulting cDNA reaction mixture (5-fold dilution) was used as a template for quantitative PCR (qPCR). The relative expression of fungal genes was estimated using the 2^−ΔΔCt^ method, as previously described [53]. The standard qPCR reaction mix was composed of 2 µL of 5× HOT FIREPol EvaGreen qPCR Mix Plus (ROX) (Solis Biodyne^®^, Tartu, Estonia), 0.3 µL of primer F (10 µM), 0.3 µL of primer R (10 µM), 15 ng of template cDNA, and water to 10 µL. Quantification values for the transcripts represent a medium with a minimum of three biological replicates and three technical repeats for each. The primers used for qPCR are listed in Table S1.

### 3.5. Estimation of F. graminearum in Barley Leaves

To determine the amount of *Fg* in inoculated barley leaves, the transcript reads of the following *Fg* housekeeping genes were analyzed: Gamma-actin (FGSG_07335) [54], GAPDH (FGSG_06257), EF1A (FGSG_08811), and B-tubulin (FGSG_09530) [52,55]. The transcript reads of each selected housekeeping gene, based on featureCounts, were compared between *Fg* inoculated barley leaves not treated with CS_10 (*Fg*) and treated with CS_10 (Fg_CS variant). The results were shown as the relative number of reads of the transcripts of each *Fg* selected gene.

## 4. Conclusions

The collection of different batches of CS showed different antifungal properties, indicating that this type of biological activity depends on the physicochemical properties of a particular CS sample. Based on the results of the microtiter and Petri plates assays, CS_10 showed the strongest effect on the growth of *Fg*. It was selected for all other studies and used at a concentration of 200 ppm. Batches of medium to high MW showed decreasing antifungal activities, clearly lower compared to CS-10. Batches of oligo chitosan with very low MW showed very weak antifungal properties, barely detectable at a concentration of 200 ppm.

The experimental time points, 48 hpi for CS treatment, 120 hpi for collection of transcriptomics, metabolomics, and for evaluation of disease symptoms, were found to be the best. HPLC-based toxin quantification showed a significant, over 4.4-fold reduction in DON and DON-3G toxins in *Fg* inoculated and CS_10 treated leaves compared to the control.

The transcriptional response of *Fg* for CS_10 treatment, analyzed in the *Fusarium*-barley leaf pathosystem revealed a significant effect on gene expression with 730 down-regulated DEGs and 297 up-regulated DEGs. In particular, among the genes strongly down-regulated, we found a set of genes involved in carbohydrate and fatty acid metabolism. Furthermore, genes of the glycolysis pathway were strongly down-regulated, possibly restricting the supply of toxin precursors and negatively affecting fungal survival and pathogenesis. Strong up-regulation of *Fg* genes involved in the oxidative stress response was observed. Surprisingly, the *Tri* genes (*Tri3*, *Tri4*, *Tri5*, *Tri7*, *Tri8*, *Tri11*, *Tri101*), which are directly related to the synthesis of trichothecene mycotoxin, were also up-regulated. 

The study indicates *Fg* genes that could be used as gene-specific targets for SIGS, HIGS, or CRISP/Cas strategies aiming at restricting pathogenesis. Furthermore, the biological properties of CS were explored, providing more information on the molecular basis of the antifungal activity of chitosan.

## Figures and Tables

**Table 1:** Characteristics of the chitosan batches used in this study.

No.	Name of the Sample	Viscosity [cps]	Molecular Weight [kDa]	Degree of Deacetylation [%]	Origin	Provider
1	CS_oligo_F	NP *	≤5	NP *	Fungal	Pol-Aura
2	CS_oligo_	NP *	≤5	≥72	Shrimp	Pol-Aura
3	CS_5	5	20	≥90	Shrimp	Glentham
4	CS_10	10	30	≥90	Shrimp	HMC+
5	CS_8-15	8–15	20–100	87.6–92.5	Shrimp	Pol-Aura
6	CS_10-120	10–120	NP *	≥85	*Aspergillus niger*	Pol-Aura
7	CS_10-100	10–100	580	≥90	Squid	Pol-Aura
8	CS_20-300	200–300	50-190	75–85	Shrimp	Sigma-Aldrich
9	CS_30-100	30–100	250	≥90	Shrimp	Pol-Aura
10	CS_100-300	100–300	890	≥90	Shrimp	Pol-Aura
11	CS_200-800	200–800	NP *	75–85	Shrimp	Sigma-Aldrich
12	CS_300-1k	300–1000	1250	≥90	Shrimp	Pol-Aura
13	CS_800-2k	800–2000	1500	≥72	Shrimp	Sigma-Aldrich
14	CS_1k-2k	1000–2000	1500	≥90	Shrimp	Pol-Aura

* NP—not provided.

**Table 2:** Significant up and down-regulated pathways in the *F. graminearum* transcriptome after chitosan CS_10 application.

Direction	Adj. *p* Value	nGenes	Pathways
Enriched Pathways in DEGs for GO Molecular Functions			
Down-regulated	3.2 × 10^−44^	77	Hydrolase activity, hydrolyzing O-glycosyl compounds
	1.4 × 10^−25^	316	Catalytic activity
	4.4 × 10^−11^	20	Carbohydrate binding
	7.4 × 10^−10^	100	Oxidoreductase activity
Up-regulated	1.4 × 10^−15^	59	Transporter activity
	4.2 × 10^−6^	17	Tetrapyrrole binding
	4.2 × 10^−6^	17	Heme-binding
	9.7 × 10^−5^	11	Monooxygenase activity
Enriched pathways in DEGs for GO Biological Process			
Down-regulated	3.5 × 10^−39^	90	Carbohydrate metabolic process
	1.8 × 10^−14^	17	Polysaccharide catabolic process
	1.8 × 10^−12^	12	Xylan metabolic process
	3.9 × 10^−12^	16	Cell wall organization or biogenesis
Up-regulated	2.9 × 10^−17^	62	Transmembrane transport
	1.5 × 10^−12^	64	Establishment of localization
	5.1 × 10^−4^	14	Ion transport
	8.4 × 10^−5^	5	Secondary metabolic process
Enriched pathways in DEGs for GO Cellular Component			
Down-regulated	4.7 × 10^−14^	20	Extracellular region
Up-regulated	3.7 × 10^−7^	48	Membrane
	9.8 × 10^−6^	31	An integral component of the membrane
	1.8 × 10^−3^	2	Fatty acid synthase complex

## Supplementary Materials

The following supporting information can be downloaded at: https://www.mdpi.com/article/10.3390/ijms241612894/s1.
