# Supplementary material for: Low Molecular Weight and High Deacetylation Degree Chitosan Batch Alleviates Pathogenesis, Toxin Accumulation, and Fusarium Gene Regulation in Barley Leaf Pathosystem
Source: Int J Mol Sci. 2023 Aug 17;24(16):12894. doi: 10.3390/ijms241612894 (PMC10454492; doi:10.3390/ijms241612894)
Supplement: Supplementary file 1 [file ijms-24-12894-s001.zip › ijms-2527053-supplementary.pdf]

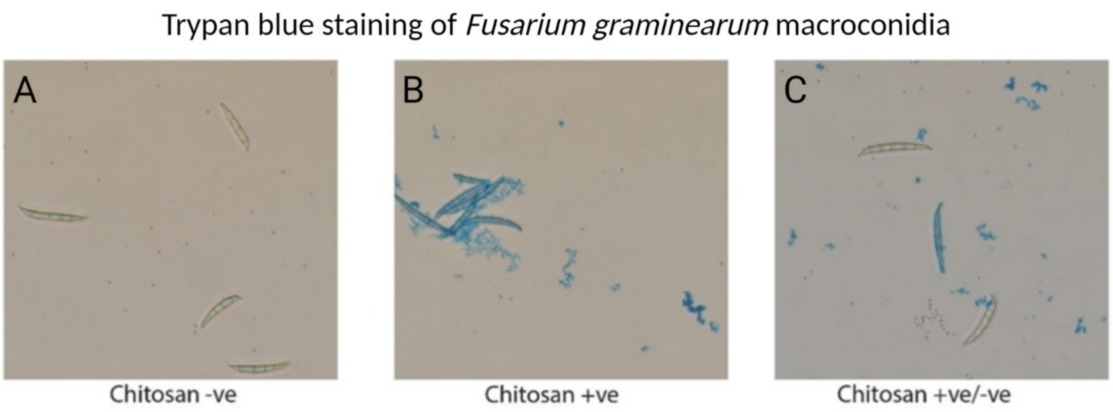

Figure S1. Light microscope picture (200x magnification) of trypan blue staining of *F. graminearum* macroconidia cultured in PDB medium with CS_10 at a concentration of 200 ppm. (A) Negative control culture (no CS_10 application) of Fg showing no staining with Trypan blue and indicates live *Fg* macroconidia. (B) Positive culture (CS_10 application) of killed and stained *Fg* macroconidia showing the antifungal impact of CS_10. (C) A mixed culture of negative and positive *Fg* macroconidia showing the authenticity of the Trypan blue staining method.

RNA-Seq Data Ouputs

Ensemble ID	Function	Log2 Fold Change	Regulation
FGSG_03842	carbohydrate metabolic process-calcium ion binding	-2.85	Down
FGSG_00571	carbohydrate metabolic process-cellulose binding	-7.52	Down
FGSG_06605	carbohydrate metabolic process	-1.49	Down
FGSG_00007	iron ion binding	1.52	Up

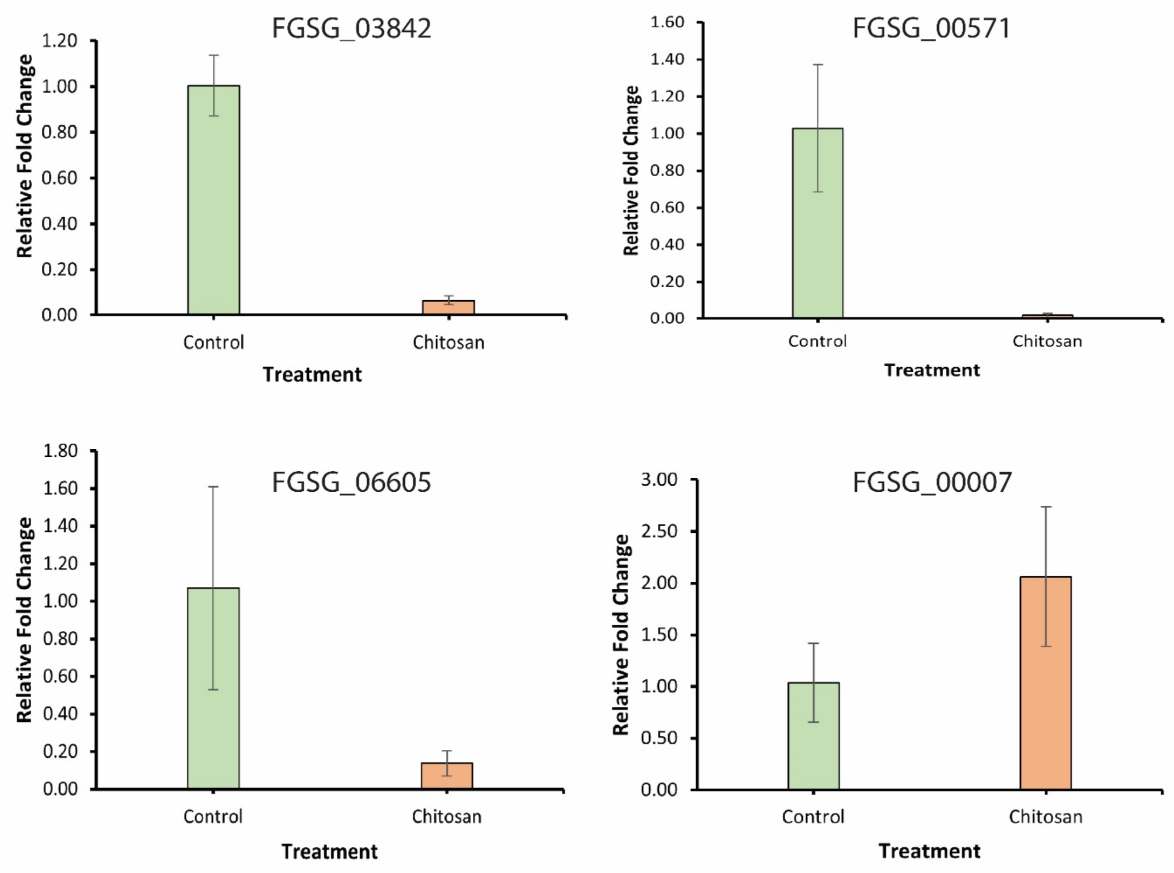

Figure S2. qPCR expression of *F. graminearum* genes in response to chitosan treatment. Relative expression levels of the *Fg* were normalized using the beta-tubulin gene as internal control and were measured through the 2−ΔΔCt method. The bar plots represent the relative fold change expression of three biological replicates and error bars represent the average means. The qPCR expression is in line with the RNA-Seq Data and confirms the validity of the expression.

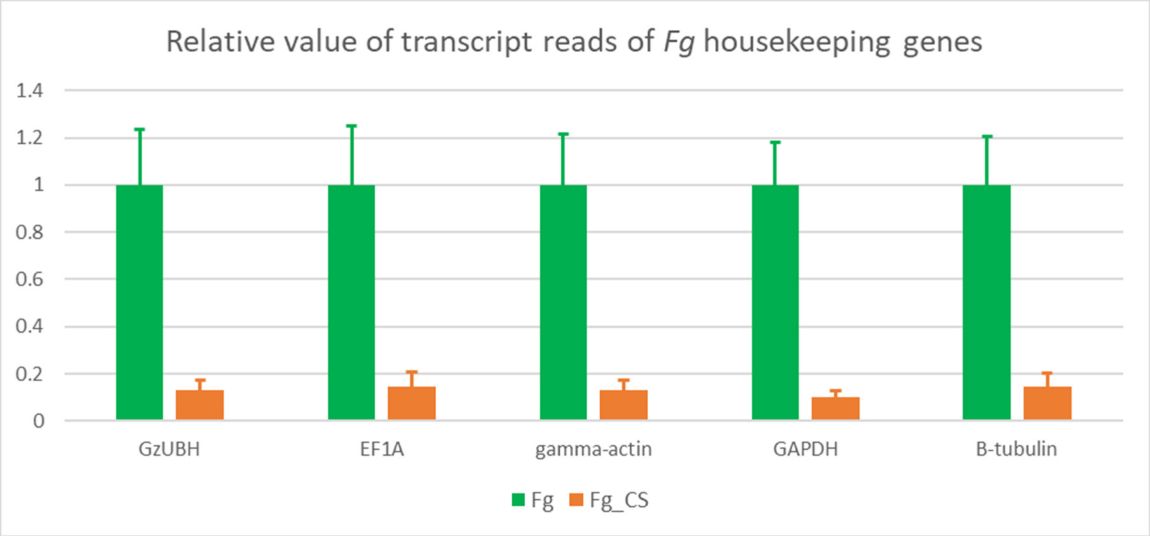

Figure S3. Relative value of transcript reads of *F. graminearum* housekeeping genes. Number of transcripts reads for each of selected housekeeping genes has been estimated based on transcriptomics results. The number of transcripts reads of the *Fg* sample has been counted as '1' while the sample treated with CS (*Fg*_CS) is shown as relative value.

Table S1: The list of Primers used for qPCR experiment based on Harris at al. (2016).

**Gene Name**	**Sequence**	**Gene involved in**
FGSG_03842	F: GAACACGGATGGTGGATACC<br>R: CCCTTTACCCATGTGGTTTG	Starch synthesis pathways
FGSG_00571	F: GGCTCCCATGGTTCTTGTTA<br>R: GTTGGGGACGTTCTTCTCAA	Starch synthesis pathways
FGSG_06605	F: TCTGGAGTCATTGCTTGTGC<br>R: GCGAAAGGCCAGTTGTAGAG	Starch synthesis pathways
FGSG_00007	F: GTGCGGTTTTGACCAGATTT<br>R: TATCAAGAGAGCGCAGCAGA	Cytochrome Synthesis
Fg-Elongation Factor 1 (FGSG_08811)	F: CCTCCAGGATGTCTACAAGA<br>R: CTCAACGGACTTGACTTCAG	Housekeeping gene (Harris, et al. 2016)

Harris, L. J., Balcerzak, M., Johnston, A., Schneiderman, D., & Ouellet, T. (2016). Host-preferential *Fusarium graminearum* gene expression during infection of wheat, barley, and maize. *Fungal Biol, 120*(1), 111-123.

Table S2. The list of differentially expressed genes discussed in the manuscript.

Gene ID	log2fold change	fold change	Annotation
FGSG_00071	1.424	2.684	TRI1
FGSG_00571	-7.522	0.005	exoglucanase 1 precursor
FGSG_01403	-0.791	0.578	minor allergen alt a 7
FGSG_01419	-1.209	0.433	long-chain acyl- synthetase
FGSG_01786	-2.273	0.207	ent-kaurene oxidase
FGSG_01959	0.277	1.212	cytochrome P450 61
FGSG_02113	-0.816	0.568	cytochrome p450 3a7
FGSG_02327	5.203	36.843	flavin adenine dinucleotide (FAD)
FGSG_02328	4.090	17.036	multicopper oxidase
FGSG_02366	1.724	3.302	sterigmatocystin biosynthesis
FGSG_02367	1.565	2.959	trichodiene oxygenase cytochrome P450
FGSG_02371	2.863	7.276	cytochrome P450 monooxygenase
FGSG_03049	-5.749	0.019	alpha-n-arabinofuranosidase 2
FGSG_03532	1.781	3.436	TRI8
FGSG_03533	1.519	2.865	TRI7
FGSG_03534	1.541	2.911	TRI3
FGSG_03535	1.390	2.620	TRI4
FGSG_03537	1.645	3.128	TRI5
FGSG_03540	1.300	2.461	TRI11
FGSG_03624	-2.485	0.179	XYLB
FGSG_03628	-6.517	0.011	exoglucanase-6A precursor
FGSG_03795	-6.930	0.008	endoglucanase 3 precursor
FGSG_03796	0.406	1.325	prostacyclin synthase
FGSG_05140	-1.372	0.386	acyl- dehydrogenase
FGSG_05551	-1.198	0.436	trans-enoyl- isomerase
FGSG_06445	-6.595	0.010	glycosyl hydrolase family 10
FGSG_06554	2.663	6.332	catalase 1
FGSG_06605	-1.491	0.356	beta-glucosidase 1 precursor
FGSG_06733	2.007	4.021	catalase 3
FGSG_07274	-2.827	0.141	probable beta-glucosidase
FGSG_07695	-6.928	0.008	xylosidase glycosyl
FGSG_07765	-2.553	0.170	trichothecene c-15 hydroxylase
FGSG_07896	1.025	2.035	TRI10
FGSG_08011	-6.584	0.010	endo-beta- -glucanase d
FGSG_08800	0.347	1.272	bzip transcription factor (Fgap1)
FGSG_09786	0.665	1.586	NADPH-cytochrome P450 reductase
FGSG_09895	-0.159	0.896	neutral trehalase
FGSG_10571	-2.089	0.235	O-methylsterigmatocystin oxidoreductase
FGSG_10571	-2.089	0.235	O-methylsterigmatocystin oxidoreductase
FGSG_10999	-6.697	0.010	XYLA
FGSG_11208	-5.928	0.016	xyloglucanase
FGSG_11258	-7.085	0.007	endo-1,4-beta-xylanase B
FGSG_11428	-5.812	0.018	feruloyl esterase B
FGSG_11656	5.596	48.370	fatty acid synthase beta subunit
FGSG_12369	1.127	2.184	catalase 4
FGSG_12551	-6.376	0.012	hypothetical protein
FGSG_13855	5.667	50.825	hypothetical protein
